# Acknowledging a Stutter Affects the Impression One Makes in a Job Interview

**DOI:** 10.1111/1460-6984.70035

**Published:** 2025-04-10

**Authors:** Jennifer Perez, Leonard S. Newman, Jenna M. Walmer

**Affiliations:** ^1^ Department of Psychology Syracuse University Syracuse New York USA

**Keywords:** disclosure, employment, speech disorders, stigma, stuttering

## Abstract

**Background:**

People who have a stutter are often viewed negatively by others. Acknowledgement—defined as notifying others up front about characteristics that might lead one to be evaluated negatively—might be an effective impression management strategy for people stigmatized by stuttering. Past research testing this hypothesis, however, has produced inconsistent findings.

**Aims:**

To assess the effectiveness of acknowledgement by people who stutter as a strategy for eliciting more positive evaluations from others while controlling for possible methodological problems in past research. Participants were expected to be more likely overall to find job applicants without a stutter to be better candidates for a job than those with a stutter. However, it was also hypothesized that this difference would not be significant when candidates with a stutter acknowledged it.

**Methods & Procedures:**

Participants watched two simulated job interview videos: in one, the applicant had a stutter, and in the other, they did not. For half of the participants, the applicant who stuttered acknowledged the speech dysfluency at the beginning of the job interview. After watching each interview, the participants rated the interviewee.

**Outcomes & Results:**

The results revealed the predicted significant interaction between stuttering and acknowledgement: acknowledgement of the stutter led the stuttering job candidate to be evaluated just as positively as the non‐stuttering job candidate.

**Conclusions & Implications:**

This research has implications for the kind of advice practitioners can offer people anticipating interactions (such as job interviews) where they will be evaluated. The findings also potentially widen the range of circumstances in which acknowledgement can be used to counteract the negative impressions people might be biased to form of stigmatized individuals.

**What This Paper Adds:**

**What is already known on the subject**

- Previous research shows that acknowledging a stigmatized identity (and other forms of acknowledgement) upfront before interacting with others in an evaluative context (such as a job interview) can often improve the impression one makes and how one's performance is perceived. However, past research evaluating this strategy in the case of stuttering has produced inconsistent findings. In addition, some past research suggests that acknowledgement might not be effective when the source of stigma is perceptually salient during the interaction.

**What this paper adds to the existing knowledge**

- The results of this study, designed to control for possible methodological problems in past research, indicate that acknowledgement can in fact be an effective impression management strategy for people who stutter.

**What are the potential or actual clinical implications of this work?**

- This research has implications for the kind of advice practitioners can offer people anticipating interactions (such as job interviews) where they will be evaluated. In short, it provides a method for trying to reduce the discrimination a person who stutters might face.

## 1 Introduction

Stuttering is a speech disorder characterized by repetitions, blocks, and prolongations of sounds and syllables. There are two types of stutters: developmental and acquired, the latter of which can be subdivided into neurogenic and psychogenic (Buchel and Sommer 2004). Developmental stuttering occurs during early childhood when speech and language are still being developed. Neurogenic stuttering is acquired due to brain injuries or neurodegenerative disorders, and psychogenic stuttering results from prolonged stress or traumatic events. Consequently, stuttering can affect people of all ages. It can also affect people in any country, culture or society, whether those people reside in Europe (Sommer et al. 2021), Asia (Ma et al. 2023), Africa (Klompas and Ross 2004) or elsewhere (see also Yairi and Ambrose 2013). SheikhBahaei and Maguire (2020) estimated that there are currently over 70 million people in the world who have a stutter.

Stuttering is also widespread in the United States, where this study was conducted. Buchel and Sommer (2004) reported an estimate of 3 million people in the country who stutter (approximately 1% of the population). More recently, Zablotsky et al. (2019) reported a prevalence rate for stuttering of 2% among American children aged 3–17 years. And although stuttering rates decrease with age, if Craig et al.’s (2002) estimates are still accurate, approximately 1 million adults aged 21–50 years stutter in the United States.

Even though there is a high prevalence of stuttering, much research reveals that the public's attitude towards those who stutter is generally negative (Boyle 2013; Johnson 2008; Klein and Hood 2004). For example, St. Louis (2020) found that public attitudes towards stuttering are nearly as negative as public attitudes towards mental illnesses. The findings from another survey were that college students perceived lawyers who had a stutter as less competent and intelligent as opposed to lawyers without a stutter (Silverman and Paynter 1990). Stigmatization of people who stutter is as worldwide a phenomenon as is stuttering itself (Arafa et al. 2021; Chu et al. 2022).

Such negative views about people who stutter have hindered people who stutter from achieving life goals. One survey found that more than 70% of people who stutter believe that their stutter lowers their chances of being employed (Klein and Hood 2004). This was also documented in a survey of employers’ attitudes towards stuttering, which found that a majority of employers held negative attitudes towards potential employees who stutter (Hurst and Cooper 1983). Employers indicated that they believe stuttering interferes with one's job performance.

Other research has focused on efforts to change attitudes towards people who stutter. For example, Flynn and St. Louis (2011) tested the hypothesis that adolescents’ attitudes towards people who stutter could be improved via an educational intervention. An oral presentation was given to high school students by a person who stuttered, and he shared anecdotes about his experience with stuttering, how he coped with having a stutter, and how stuttering had impacted his life. The presentation significantly reduced negative attitudes towards people who stutter. The results of the study demonstrated that such interventions have the potential to reduce the stigma associated with stuttering.

What, though, can individuals who stutter do to affect how they are perceived by others in the situations in which they find themselves, other than hoping that public attitudes will change? The current study tests the hypothesis that people might be able to at least partially counteract the stigma they often experience by means of *acknowledgement*. By acknowledgement, we mean people explicitly notifying others up front about characteristics that might lead them to be perceived negatively (in this instance) and/or factors that might inhibit their performance. Acknowledgement has been shown to be effective for eliciting more positive evaluations from others in a number of situations (Ward and Brenner 2006). For example, Ward and Brenner^1^ found that when a professor addressed having a strong accent before giving a lecture, participants rated his speech as being clearer. Another example that supports the effectiveness of acknowledgement was the finding that when a college applicant acknowledged they did not have the greatest grades, participants rated them more favourably in terms of their suitability for college admission (Ward and Brenner 2006). Acknowledgement can also improve a consumer's rating of a product or service. This was illustrated in a research study that examined whether a restaurant could improve how people rated it by acknowledging that their menu was small and limited (Pfeiffer et al. 2014). The researchers found that addressing the negative quality of the restaurant's menu resulted in more positive ratings from participants. As seen in both of these research studies, acknowledgement can improve overall attitudes in various situations.

Of even more relevance to the present investigation, acknowledgement has been found to be an effective impression management tactic for people with socially stigmatizing characteristics. In a number of studies conducted by Hebl and colleagues involving mock interviews (Hebl and Kleck 2002; Hebl and Skorinko 2005) or real ones (Singletary and Hebl 2009), outcomes for interviewees who openly acknowledged and explicitly commented on their obesity, physical disability, or sexual minority status were compared to those of similarly stigmatized interviewees who did not do so. For example, half of the interviewees in Hebl and Kleck (2002) stated that ‘When people meet me, one of the first things that they notice is that I'm overweight’ or ‘I use a wheelchair’. In Singletary and Hebl (2009), female job applicants wore hats on with either the slogan ‘Texan and Proud’ or ‘Gay and Proud’. Half of the interviewees also stated, ‘I don't usually wear relaxed hats like this, but this is a good reflection of who I am’. The results of these studies were that stigmatized individuals who engaged in acknowledgement were evaluated more positively than those who did not.

Given those findings, it might seem reasonable to simply assume that acknowledgement should be an equally effective means of impression management for people with verbal dysfluencies such as a stutter. However, a number of considerations caution against making that assumption. One is a distinction made by Madera and Hebl (2012) to help account for the results of a study that failed to find a significant effect for acknowledgement. Madera and Hebl had participants observe simulated interviews in which, half the time, interviewees had a facial stigma—specifically, either a port‐wine stain or a scar on one cheek. Acknowledging the facial blemish had no effects on the impressions observers formed of the stigmatized individuals. Madera and Hebl speculated that

normal face processing involves visual attention to the eyes, nose, and mouth (Li and Jain 2005). Given that the facial stigma was in such close proximity to these other areas of visual attention, acknowledgement may simply not have effectively reduced attention to the facial stigma (324).

By analogy, normal speech processing involves auditory processing of the voice. Acknowledgement might not be sufficient for reducing attention to a speech dysfluency and might not be enough to counteract the negative influence it might have on how a listener perceives the interviewee.

A more direct reason for questioning the effectiveness of using acknowledgement to manage the impressions people form of individuals who stutter is that the results of past investigations that have assessed it have been mixed. Some have found that it makes a difference, and some have not (Byrd, McGill et al. 2017; Collins and Blood 1990; Healey et al. 2007; Lee and Manning 2010; Lincoln and Bricker‐Katz 2008), and even within studies, the results are often quite mixed (e.g., Croft and Byrd 2021). The current investigation was designed to control for some of the methodological issues that might have clouded the results of past studies. In some of them, participants were presented with the same target person twice, once acknowledging a stutter and once not (Byrd, McGill et al. 2017; Lee and Manning 2010). In others, the focus of the researchers on the issue of stuttering was explicitly stated (Collins and Blood 1990), or no cover story seems to have been provided to participants to provide them with a reason for why they were being asked to evaluate the target persons (Byrd, Croft et al. 2017; Healey et al. 2007; Lee and Manning 2010; Lincoln and Bricker‐Katz 2008). Both of these features of past studies increase the likelihood that participants could have intuited the purpose of the studies and/or the hypotheses, thereby introducing possible demand effects (Nichols and Maner 2008). The current study employed a cover story to reduce suspiciousness.

Additionally, some past studies included only one stuttering person as a target for participants’ ratings (Byrd, Croft et al. 2017; Lee and Manning 2010; Lincoln and Bricker‐Katz 2008; Werle et al. 2023). Use of just one exemplar to represent a conceptual category can make it more difficult to generalize from the results of a study (see Wells and Windschitl 1999 on stimulus sampling). In the current study, participants rated one of two people who stuttered.

Finally, most past studies did not include a non‐stuttering condition. Thus, although they were designed to answer the question ‘Can acknowledgment of a stutter lead other people to form a more positive impression of the person who stutters?’ they could not answer a question that is at least equally as important: could the effect of acknowledgement be powerful enough that the impressions formed of people who stutter would be *no different than they would have been if those people had not stuttered at all*? The current study was designed to address that question.

This research project, then, was designed to provide another test of the hypothesis that acknowledgement of speech disorders can elicit more positive evaluations from others. The specific setting that was examined was a (mock) job interview. The results of the study could inform people with speech dysfluencies about how they could best present themselves in job interviews and other similar situations in everyday life. The first prediction was that participants would be overall more likely to find job applicants without a stutter to be better candidates for a potential job than those with a stutter. However, it was also hypothesized that this difference would not be significant when candidates who stuttered acknowledged their speech dysfluency. An interaction between stuttering (present versus absent) and acknowledgement of stuttering (yes versus no) was therefore predicted.^2^

## 2 Method

### 2.1 Participants

A total of 58 participants were recruited for this research study; G*Power 3.1 indicated that this sample size would be sufficient for detecting effects of the sizes reported by Ward and Brenner (2006) in their studies of acknowledgement. Participants were current undergraduate students registered for their university's psychology department subject pool and were at least 18 years of age. Students who participated signed up via the department's online study management system, and they received a half hour of credit that was counted towards a requirement for their introductory psychology class. All students provided written consent to participate in the research.

There were 24 male participants and 34 female participants. The majority of the participants’ first language was English (82.8%, *n* = 48). Participants ages ranged from 18 to 23 years, with a mean age of 18.9 years. The majority of the sample was White/Caucasian (60.3%, *n* = 35), followed by Asian (15.5%, *n* = 9), Hispanic/Latino (13.8%, *n* = 8), Black/African American (6.9%, *n* = 4) and Other (3.4%, *n* = 2). Five (four male, one female) participants reported having a stutter. These participants were excluded in the analyses presented below (although all significant effects remained significant when they were included, and the pattern of means remained the same).

All studies were reviewed and approved by a university institutional review board (IRB), and participants in all studies provided informed consent to participate in the research after reading consent forms approved by the IRB.

### 2.2 Materials and Procedure

Two actors were recruited to participate on a volunteer basis in the simulated job interviews for this study. Both actors were undergraduate students at the university where the research was conducted. The female actress was job applicant 1 (viewed first), and the male actor was job applicant 2 (viewed second) in the videos shown to the participants. The actors were provided with a script to read from in the filmed video; for the interview scripts, see Appendix A. The first author served as the interviewer. The job interview videos were recorded via Zoom. Both actors, filmed from the shoulders up, acted out three different job interview scenarios. In the first video filmed, the actor spoke with no stutter. In the second video filmed, the actor spoke with a stutter without acknowledging it. In the third video filmed, the actor spoke with a stutter and acknowledged it (both actors were trained by the first author, a research assistant in a speech production research laboratory). Healey et al. (2007), Hebl and Skorinko (2005) and Lincoln and Bricker‐Katz (2008) found that acknowledgement was most effective when it occurred at the beginning (as opposed to the later stages) of a social interaction. Thus, the actor acknowledged the stutter in a non‐apologetic manner (Byrd, Croft et al. 2017) at the beginning of the interview by stating, ‘Just to let you know I have a stutter’. The job interview videos were viewed by the participants via a computer in a research lab.

Up to two participants could be run in each session, but they were run either singly or in pairs. When run in pairs, there was no discussion between them. Participants watched two brief videotaped job interviews (just 2–3 min each), and they were told that the purpose of the research study was to test out a new kind of rating form that could be used to evaluate individuals undergoing an employment interview. In one interview seen by each participant, the interviewee had a stutter; in the other, they did not. For half of the participants, the interview with the stutter was seen first, and for half, it was seen second. And for half of the participants, the interviewee with the stutter acknowledged their stutter at the beginning of the interview. The condition in which participants were run (i.e., the pair of videos viewed) in a given session was randomly assigned.

After watching each interview, the participant was presented with an interview evaluation form that contained four Likert‐type survey questions, all of which were paired with a 7‐point scale (1 = Very Poor, 7 = Very Good). There were two filler questions: ‘What was the quality of the picture and sound of the video you just saw?’ and ‘How likely do you think this applicant would be to accept a job with your organization?’ These questions were included to reduce participants’ suspicions about the actual purpose of the study and so they would not be overly focused on the questions that were of main interest. The two questions relevant to the hypotheses in the interview evaluation form—those directly pertaining to how the job applicant performed in the interview—focused on the participant's overall impression of the job applicant: ‘If you were making the hiring decision, how likely would you be to offer the applicant the job?’ and ‘How good of an impression do you think the applicant made in this interview?’

The design and procedure allowed for an analysis of the data by means of analysis of variance (ANOVA) with follow‐up simple effects analyses (Keppel and Wickens 2004). This analytic procedure would allow for tests of whether (1) stuttering affected the impressions formed of the job applicants, (2) acknowledgement of the stutter affected the impressions formed of the applicants who stuttered, and (3) whether job candidates who acknowledged the stutter were evaluated any differently than those candidates who did not stutter at all.

After participants completed the form for the second interview, demographic information was collected, after which participants were debriefed. The demographic questions consisted of 6 multiple‐choice and fill‐in‐the‐blank items (e.g., ‘What is your gender?’). The demographic data collected included the participants’ gender, age, first language, and race. Participants were also asked whether they themselves had a stutter. After debriefing, participants were asked (once they knew the purpose of the study) whether the researchers could retain their data for analysis or whether they preferred to withdraw from the study. Only one participant withdrew (for unknown reasons).

## 3 Analysis and Results

Responses to the two key questions (‘If you were making the hiring decision, how likely would you be to offer the applicant the job?’ and ‘How good of an impression do you think the applicant made in this interview?’) were highly correlated, both for the first interview participants viewed (the female job applicant), *r* (53) = 0.72, *p* < 0.001, and for the second interview (the male job applicant), *r* (58) = 0.80, *p* < 0.001. The redundancy between the two variables suggested that they were both indexing global evaluation of the interviewee. Thus, for the purposes of the primary analyses, the mean of the two ratings was used as the dependent variable.

There were two independent variables in this study: acknowledgement condition was a between‐subjects variable (each participant viewed an applicant with a stutter who either did or did not acknowledge it), and stuttering was within‐subjects (each participant viewed two applicants, one who stuttered and one who did not). Thus, the variables were analysed with a 2 (acknowledgement condition: yes, no) × 2 (stuttering: yes, no) mixed‐model ANOVA.

The condition means are presented in Table 1. The first prediction was that participants would be overall more likely to find job applicants without a stutter to be better candidates for a potential job than those with a stutter. The results of the ANOVA were consistent with that hypothesis. There was a main effect for stuttering, *F*(1, 51) = 10.53, *p* = 0.002, *η_p_* ^2^ = 0.17, and as expected, the job applicant with a stutter was rated more negatively overall. There was no main effect of acknowledgement condition, nor was one predicted (as this variable collapsed across the two speakers, both the one who stuttered and the one who did not).

**TABLE 1:** Mean global evaluation of the interviewees (combination of impression and willingness to hire) by stuttering (no stutter, stutter—within subjects) and acknowledgement of stutter (yes, no—between subjects).

	No stutter	Stutter
No acknowledgement by the applicant with a stutter *(n* = 29)	5.71	4.38
	SD = 1.00	SD = 1.56
Acknowledgement by the applicant with a stutter *(n* = 24)	5.02	5.08
	SD = 1.18	SD = 1.41

*Note*: Ratings could range from 1 (very poor) to 7 (very good).

It was also hypothesized that the difference in how applicants with and without a stutter were evaluated would be lower in magnitude when the applicants who stuttered acknowledged their speech dysfluency. A significant interaction between stuttering (present versus absent) and acknowledgement of stuttering (yes versus no) was therefore predicted, and it was found: *F*(1, 51) = 12.71, *p* < 0.001, *η_p_* ^2^ = 0.20.^3^ ANOVA analyses of each question separately (job offer, impression) revealed the same significant effects as the analysis of the aggregated variable, with near‐identical patterns of means (Tables 2 and 3).

**TABLE 2:** Mean ratings of the impressions created by stuttering (no stutter, stutter—within subjects) and acknowledgement of stutter (yes, no—between subjects).

	No stutter	Stutter
No acknowledgement by the applicant with a stutter *(n* = 29)	5.76	4.38
	SD = 1.18	SD = 1.66
Acknowledgement by the applicant with a stutter *(n* = 24)	5.25	5.50
	SD = 1.36	SD = 1.29

*Note*: Ratings could range from 1 (very poor) to 7 (very good).

**TABLE 3:** Mean ratings of the likelihood of hiring candidate by stuttering (no stutter, stutter—within subjects) and acknowledgement of stutter (yes, no—between subjects).

	No stutter	Stutter
No acknowledgement by the applicant with a stutter (*n* = 29)	5.66	4.38
	SD = 0.97	SD = 1.59
Acknowledgement by the applicant with a stutter *(n* = 24)	4.80	4.67
	SD = 1.18	SD = 1.76

*Note*: Ratings could range from 1 (very poor) to 7 (very good).

To further clarify the nature of the interaction, simple effects analyses (Keppel and Wickens 2004) were conducted. These analyses revealed that when the stutter was not acknowledged, the job applicants with a stutter were rated more negatively than those who did not stutter, *F*(1, 51) = 25.61, *p* < 0.001, *η_p_* ^2^ = 0.33. On the other hand, when the stutter was acknowledged, there was no difference in how the stuttering and non‐stuttering applicants were evaluated, *F*(1, 51) = 0.05, *p* = 0.83, *η_p_* ^2^ = 0.00. While the difference between the stuttering job candidates who did and did not acknowledge their speech dysfluency was only marginally significant, *F*(1, 51) = 2.92, *p* = 0.09, *η_p_* ^2^ = 0.05, for the impression question alone (acknowledgement: *M* = 5.50, SD = 1.29; no acknowledgement: *M* = 4.38, SD = 1.66), the difference was significant, *F*(1, 51) = 7.33, *p* = 0.01, *η_p_* ^2^ = 0.13.

Order of presentation of the interviews (i.e., whether the job candidate with the stutter was presented first or second) had no effect on ratings, nor did this variable interact with any of the others (all *p*s > 0.31).

## 4 Discussion

The purpose of this study was to build on previous research investigating whether acknowledgement would improve the impressions people formed of a person who stutters. It was designed to control for methodological issues (e.g., possible demand effects) that complicated the interpretation of findings from past studies. The data indicated that mock job applicants with a stutter were overall rated more negatively than the job applicants without a stutter. As hypothesized, however, participants rated a job applicant with a stutter more positively when the stutter was neutrally acknowledged at the beginning of the job interview. As a result, when job applicants acknowledged their stutter—and only then—they received ratings equivalent to those of the job applicant without a stutter. Therefore, the data support the hypothesis that acknowledgement of a stutter would improve people's perception of how well a stuttering job candidate performed in an interview. In addition, they provide evidence that acknowledging a stutter even has the potential to undo the negative effects that a speech dysfluency can have on how an individual is evaluated.

The results of a previous study by Madera and Hebl (2012) involving individuals with a facial disfigurement suggested that when a stigmatized individual is the focus of visual attention, acknowledgement might not reduce the negative influence of this perceptually salient source of stigma. The results of the present study indicate that it may be premature to reach that conclusion. Indeed, Madera and Hebl also noted that another explanation for their null results could have been that ‘the particular wording applicants used in their acknowledgments was not powerful enough to reduce the visual attention’ (324). There are, of course, many other differences between speech dysfluencies and facial disfigurements (and other visual triggers of stigma) that could account for the different results of the two studies. Either way, future research expanding the range of stigmatizing conditions people acknowledge in evaluative contexts will hopefully clarify this issue.

An important feature of the experimental procedure was that acknowledgement of a speech dysfluency was provided by the person who stuttered at the beginning of the interaction with the interviewer. Past research has revealed that early disclosure of a stutter is a significantly more effective way to influence listener perceptions than later disclosure (Healey et al. 2007; Lincoln and Bricker‐Katz 2008). Although we can only speculate, the importance of this variable suggests that the processes at play in this study were the same ones underlying the effects of other studies that involve alerting people to possible biases in advance of a social interaction. Advance warnings about possible bias provide people with the opportunity to control how they categorize, interpret, and encode another person's behaviour (Baumeister and Newman 1994). When people attempt to correct for biases after they have already processed social information, they tend to be less effective in doing so (Gilbert and Osborne 1989; Pratkanis et al. 1988).

In sum, the results of this study suggest that acknowledgement can lead to more positive impressions of people with a stutter. Acknowledgement may be a method that people with speech dysfluencies could effectively utilize to better present themselves in job interviews and other similar interpersonal situations in everyday life.

### 4.1 Limitations

However, there were some limitations to the study. One is that the sample was not representative of the general population since the researchers only recruited college students. Therefore, the participants may have rated the job applicants who stuttered more positively because they felt more empathy towards the interviewees, as they appear to be around the same age as the participants themselves. It is also important to note that the job applicants were portrayed by actors who do not have a stutter in real life. Consequently, despite efforts to ensure that the speech dysfluencies sounded realistic, they might have differed in some subtle way from those of people who genuinely stutter.

### 4.2 Future Research and Conclusions

Few studies have directly examined the mechanisms mediating the effect of acknowledgement of socially stigmatizing characteristics on person perception and impression formation. One exception is Hebl and Skorinko (2005), which revealed that people who openly and explicitly acknowledge their disabilities and limitations are perceived to be more well‐adjusted and psychologically healthy than those who do not. Future research could provide converging evidence for that finding by testing whether changes in listeners’ inferences about the characteristics of individuals who acknowledge their speech dysfluencies (including but not restricted to adjustment and psychological health) play a significant role in their perceptions and evaluations of those individuals.

Saal et al. (2014) focused on another possible mediating mechanism. They noted that people without disabilities may find it difficult to interact with individuals with disabilities ‘because they are not sure what they should say, what they should avoid saying, whether they should acknowledge or provide assistance, or any number of other intruding and distracting thoughts’. The result could be ‘an unmanageable cognitive load’ (both 243). Acknowledgement of a disability could serve to ‘“break the ice” by releasing others from this cognitive cycle’ (245), and the reduction of stress could lead those others to be more favourably disposed towards the stigmatized individual. However, the only study on the topic to directly assess variables related to cognitive capacity was the one by Madera and Hebl (2012), and as previously mentioned, they failed to find significant effects of acknowledgement. As a result, they could not assess available cognitive capacity's role as a possible mediating variable. Future research could do so, however.

Further research is necessary to reveal the extent to which the effects of acknowledgement found in this study generalize to other situations (professional, academic, social) in which people who stutter might want to manage the impressions others form of them. In addition, future studies on acknowledgement of speech disorders should recruit a more diverse sample so that the results may be more confidently generalized. Overall, there needs to be more research on the possible outcomes of using acknowledgement in general and on its utility for people who stutter in particular. The results could help people better cope with the challenges they face on an everyday basis due to their speech disorders.

## Conflicts of Interest

The authors declare no conflicts of interest.

## Data Availability

The data file is available upon request.

## Endnotes

1 Ward and Brenner (2006) labelled this strategy ‘negative acknowledgement’. We prefer simply to refer to it as ‘acknowledgement’, so as not to imply that stutters or other speech dysfluencies are necessarily unfavourable characteristics or that they should be perceived as such.

2 Hebl and Kleck (2002) also found that acknowledgement was ineffective when perceivers believed that the stigmatized individuals were responsible for their condition (e.g., that they became physically disabled because they ‘did not choose to have a surgery that doctors felt was urgent’; 239). It is unlikely, however, that people believe that the onset of stuttering results from controllable factors such as carelessness, lack of self‐discipline or making poor choices.

3 Although this experiment was not powered a priori to detect interactions involving participant gender, the interaction between stuttering and acknowledgement condition was significant for both male and female participants, and gender did not interact with the other two independent variables (*p* = 0.38).

## Appendix A

### Interview Scripts

Job Applicant 1

Applicant: Good afternoon, I'm here for the interview.

Interviewer: Welcome, please take a seat. Thank you for your job application. I have read through your resume and have a few questions to ask you. Can you tell me a little about yourself?

Applicant: Sure! Let me start by taking this opportunity to thank you for making time to meet with me this afternoon. My name is ______ and I received my bachelor's degree in business last year from ______ University. I am interested in pursuing a career in advertising or marketing. I love to write and worked for my college newspaper. I like to write articles on traveling and fashion.

Interviewer: What are some of your weaknesses?

Applicant: Well, I would say that I am a very structured person. So, when something comes up that could switch my plans, I may stress out. However, I have learned how to plan ahead for possible problems by creating a backup plan!

Interviewer: I admire how you have learned from your weaknesses. Can you tell me more about your strengths?

Applicant: I'm a really great listener. I believe that listening skills have more value in a conversation than speaking skills. I am also a good organizer. I like to keep my space neat and organized.

Interviewer: Tell me about a recent job or volunteer experience you had.

Applicant: My last job was working in a retail store. I was responsible for organizing product displays and storage. I would count inventory, add price tags to products, and organize products on shelves. While I enjoyed this job, there was a lack of opportunity in the way I wanted to progress and succeed in my future career.

Interviewer: Why do you want to work here?

Applicant: I'm very interested in your organization and services, so I would be excited to come here and grow my skills with an organization like yours.

Interviewer: Do you have any questions for me?

Applicant: I don't think I have any. Thank you for the interview. It was a pleasure meeting you today.

Interviewer: No problem. Have a great day and take care!

Job Applicant 2

Applicant: Good afternoon, I'm here for the interview.

Interviewer: Welcome, please take a seat. Thank you for your job application. I have read through your resume and have a few questions to ask you. Can you tell me a little about yourself?

Applicant: Of course. My name is ______ and I received my bachelor's degree in biology last year from ______ University. I am interested in pursuing a career in medicine. In college, I was a part of an intramural volleyball team.

Interviewer: What are some of your weaknesses?

Applicant: I sometimes act too quickly when in a stressful situation. I have learned to act less impulsively, so I won't rush into things without thinking them through.

Interviewer: I admire how you have learned from your weaknesses. Can you tell me more about your strengths?

Applicant: I'm very collaborative and have always preferred working in groups. In my college experience, I have always excelled in group projects and ensuring work is delegated fairly. Through open communication and working with people's strengths in collaboration, I have succeeded in my group projects.

Interviewer: Tell me about a recent job or volunteer experience you had.

Applicant: I worked in the food industry. I would sometimes serve food or work in the kitchen. I really loved working as a server because I was able to meet and interact with a lot of people.

Interviewer: Why do you want to work here?

Applicant: I've researched this job and think that my skills and interests fit the description for this position. I have great attention to detail, am good at organizing and planning, and am open to learning new skills. I believe that I would be a great match for your company.

Interviewer: Do you have any questions for me?

Applicant: I do not have any. Thank you very much.

Interviewer: No problem. Have a great day, goodbye!
